# Barium Levels in Brazil Nuts: A Global Review of the Literature

**DOI:** 10.1111/1750-3841.70643

**Published:** 2025-10-31

**Authors:** Christian Koeder, Markus Keller

**Affiliations:** ^1^ Institute for Prevention and Cancer Epidemiology, Medical Center – University of Freiburg, Faculty of Medicine University of Freiburg Freiburg Germany; ^2^ Research Institute for Plant‐Based Nutrition (IFPE) Biebertal Germany

## Abstract

Brazil nuts are often recommended for their high selenium content but have also been reported to contain elevated levels of barium, a potentially toxic element. Regular daily consumption of Brazil nuts may exceed the currently suggested tolerable daily intake (TDI) for barium (ca. 0.2 mg/kg body weight/day). No comprehensive review of barium levels in Brazil nuts has been published to date. Therefore, a global literature review of barium in Brazil nuts without language or time restrictions was conducted. A total of 24 eligible publications (1933–2023) were included. The literature review indicated a mean barium level of 1.27 (95% confidence interval: 0.89, 1.64; range: 0.03–5.89; *n* = 43) mg/g. For most regions, consuming two Brazil nuts per day does not exceed the TDI for adults. However, in some regions (Germany, Guyana, and Japan), and for individuals with a body weight of <60 kg, consuming two Brazil nuts per day may already exceed the TDI, not accounting for other dietary sources of barium. Studies with georeferencing are needed to clarify geographic variability, and human studies on barium bioavailability from Brazil nuts are warranted.

## 1 Introduction

Brazil nuts (*Bertholletia excelsa*) are exceptionally rich in selenium and are frequently recommended as a natural dietary source of this element (Koeder and Perez‐Cueto 2022). However, Brazil nuts also contain high levels of the potentially toxic element barium (Martins et al. 2012; Parekh et al. 2008), with reported mean levels in the range of 1–4 mg/g (Cardoso et al. 2017; Lemire et al. 2010; Martinez‐Morata et al. 2023; Peana et al. 2021; Rayman 2008). Much lower barium levels have been reported for almonds and peanuts (ca. 0.004–0.005 mg/g) (González‐Weller et al. 2013; Pearson and Ashmore 2020) and pecans (ca. 0.007 mg/g) (WHO 2016). Barium is present in nearly all foods. Relevant dietary sources include grains and grain products (ca. 0.00–2.23 mg/kg), fats and oils (ca. 0.03–3.22 mg/kg), legumes (ca. 0.16–1.13 mg/kg), fruit (ca. 0.03–0.73 mg/kg), vegetables (ca. 0.05–1.13 mg/kg), cheese (ca. 0.34–0.95 mg/kg), chocolate (ca. 1.69–4.36 mg/kg) (Jaćimović et al. 2022), chicken eggs (ca. 0.37–0.57 mg/kg), wine (ca. 0.00–0.39 mg/L) (González‐Weller et al. 2013; Rose et al. 2010), soft drinks (ca. 0.03–0.20 mg/L) (González‐Weller et al. 2013), and plant‐based protein supplements (ca. 1.04–2.70 mg/kg) (Bethencourt‐Barbuzano et al. 2025).

Barium has no known biological function in humans (Peana et al. 2021). Chronic high intake may impair kidney function and the nervous and cardiovascular systems (Kowalczyk et al. 2022; Peana et al. 2021). Possible bone‐protective effects have also been suggested (Coyte et al. 2022). Acute barium toxicity can cause abdominal pain, vomiting, diarrhea, paresthesia, muscle cramps or weakness, blood pressure changes, respiratory paralysis, ventricular tachycardia (Ghose et al. 2009; Kowalczyk et al. 2022; Moraes de Brito et al. 2019), and hypokalemia (sometimes severe) (Payen et al. 2011; Rayman 2008).

Food is the primary source of non‐occupational barium exposure for most humans (Kravchenko et al. 2014). Barium concentrations in Brazil nuts vary widely (Armelin et al. 2019; Hiromoto et al. 1996; Smith 1971). Although barium levels in Brazil nuts have been reported since the 1930s (Seaber 1933; Wagner 1936), no global review exists. Such a review is needed to assess whether barium in Brazil nuts poses a risk and if intake should be limited.

The present comprehensive literature review aims to summarize existing data on barium levels in Brazil nuts and proposes acceptable upper intake levels.

## 2 Literature Search Strategy

The terms “Brazil nuts,” “Brazilian nuts,” “nuts,” “Bertholletia,” “barium,” “minerals,” and “elements” were used to search PubMed, Semantic Scholar, and Web of Science. The search concluded on May 1, 2025. Titles and references of identified articles were screened to identify studies reporting barium levels in Brazil nuts. No language restrictions were applied to journal articles.

## 3 Statistical Analysis

To calculate mean barium levels from the literature, subgroup‐level data (e.g., by federal state, shelled/in‐shell, wild‐collected/store‐bought (Armelin et al. 2019)) were treated as independent and equally weighted (subgroup‐level means). Sensitivity analyses included weighting means by publication (publication‐level means) and excluding studies on defatted nuts. Results are reported as mean ± standard error of the mean. Analyses were performed using IBM SPSS Statistics (Armonk, NY).

## 4 Publications Identified in the Literature Review

Twenty‐five publications reported original barium concentrations in Brazil nuts (Andrade et al. 1999; Armelin et al. 2019; Berenguel et al. 2018; da Silva Junior et al. 2022; Frank and Betancourt 1980; Furr et al. 1979; Gonçalves et al. 2009; Hiromoto et al. 1996; Kobashi and Tominaga 1985; Lemire et al. 2010; Lisk et al. 1988; Moraes de Brito et al. 2019; Moreda‐Piñeiro et al. 2016; Naozuka et al. 2011; Parekh et al. 2008; Penna‐Franca et al. 1968; Rodushkin et al. 2008; Rovasi Adolfo et al. 2021; Seaber 1933; Silva Duarte et al. 2023; Smith 1971; Stoewsand et al. 1988; Wagner 1936; Welna et al. 2008; Welna and Szymczycha‐Madeja 2014) (Table 1). However, one study reported only a barium range (Hiromoto et al. 1996). With the exception of one study using nuts from a botanical garden in Singapore (Smith 1971), all Brazil nuts in the present review can be assumed to have originated from the Amazon rainforest. Apart from studies on Brazil nuts obtained directly in Brazil (Seaber 1933) and Guyana (Smith 1971), no studies of Brazil nuts directly obtained in other Latin American countries were identified. Two studies investigated Brazil nuts obtained in Asia (Singapore [Smith 1971] and Japan [Kobashi and Tominaga 1985]). No studies from Africa were identified. Mean barium concentrations from the literature are shown in Table 1. Additional information on included and excluded studies is presented in Supporting Information 1.

**TABLE 1:** Barium concentrations in Brazil nuts from the literature, including original data.

Publication	Nut description	Obtained in	Origin	Barium (mg/g)	
				Mean	SD
Silva Duarte et al. (2023)	—	BR	*Amazonas*	0.05	0.01
da Silva Junior et al. (2022)	Gathered	BR	*Acre*	2.73	2.43
			*Amapá*	0.42	0.30
			*Amazonas*	0.05	0.02
			*Pará*	0.03	0.02
			*Rondônia*	0.18	0.16
			*Roraima*	0.77	0.35
Rovasi Adolfo et al. (2021)	—	BR (*Rio Grande do Sul*)	BR (assumed)	0.82	0.03
Armelin et al. (2019)	Gathered	BR	BR	0.03	NA
	Store‐bought			1.97	NA
Berenguel et al. (2018)	—	BR	BR	1.53	0.09
Moraes de Brito et al. (2019)	Shelled	BR (Belém, *Pará*)	BR	0.31	0.25
Moreda‐Piñeiro et al. (2016)	—	ES	NA	1.38	0.04
Welna and Szymczycha‐Madeja (2014)	Defatted	PL	NA	2.21	0.15
Naozuka et al. (2011)	—	BR (assumed)	*Pará*	0.47	0.01
Lemire et al. (2010)	—	BR (*Pará*)	*Pará*	0.09^a^	NA
Gonçalves et al. (2009)	—	BR (*Maranhão*)	BR	1.82	0.06
	—	BR (*São Paulo*)		2.08	0.06
	In shell	BR (*Pará*)		0.90	0.01
	In shell, in capsule	BR (*Pará*)		0.88	0.03
	Shelled	BR (*Pará*)		1.93	0.04
Parekh et al. (2008)	—	US	BR	0.74	0.01
			BO	1.99	0.02
			PE	0.56	0.01
			NA	0.10	0.01
Rodushkin et al. (2008)	Shelled	SE	NA	0.38	0.06
Welna et al. (2008)	In shell	PL	NA	0.07	0.01
Andrade et al. (1999)	—	BR (Belém, *Pará*)	BR	1.51	NA
Hiromoto et al. (1996)	—	BR	BR	[0.6–3.1]	
Lisk et al. (1988)	—	US	NA	1.95	NA
Stoewsand et al. (1988)	In shell	US	NA	1.00	NA
Kobashi and Tominaga (1985)	Imported from the US	JP	NA	4.37	0.03
Frank and Betancourt (1980)	Farmed	DE	BR (*Amazonas*)	0.08	NA
	Gathered			2.92	NA
				1.80	NA
				1.67	NA
Furr et al. (1979)	—	US	NA	1.71	NA
Smith (1971) (UK study)	—	GY	GY (Dadanawa)	0.53	NA
			GY (Rewa)	5.89	NA
		SG	SG	0.33	NA
				0.19	NA
Penna‐Franca et al. (1968)	—	BR	BR	1.76	NA
Wagner (1936)	In shell	DE	NA	2.6	NA
Seaber (1933)	—	BR (UK study)	BR	1.74	0.89

*Note*: Brazilian states appear in italics. Values in square brackets are supplementary and were excluded from calculations (see main text). The terms “in shell” and “shelled” refer to nuts obtained (not analyzed) with the shell intact or removed, respectively.

Abbreviations: BO, Bolivia; BR, Brazil; DE, Germany; ES, Spain; GY, Guyana; JP, Japan; NA, not available; PE, Peru; PL, Poland; SD, standard deviation; SE, Sweden; SG, Singapore; UK, United Kingdom; US, United States of America.

^a^Median.

## 5 Barium Levels in Brazil Nuts

**FIGURE 1:**
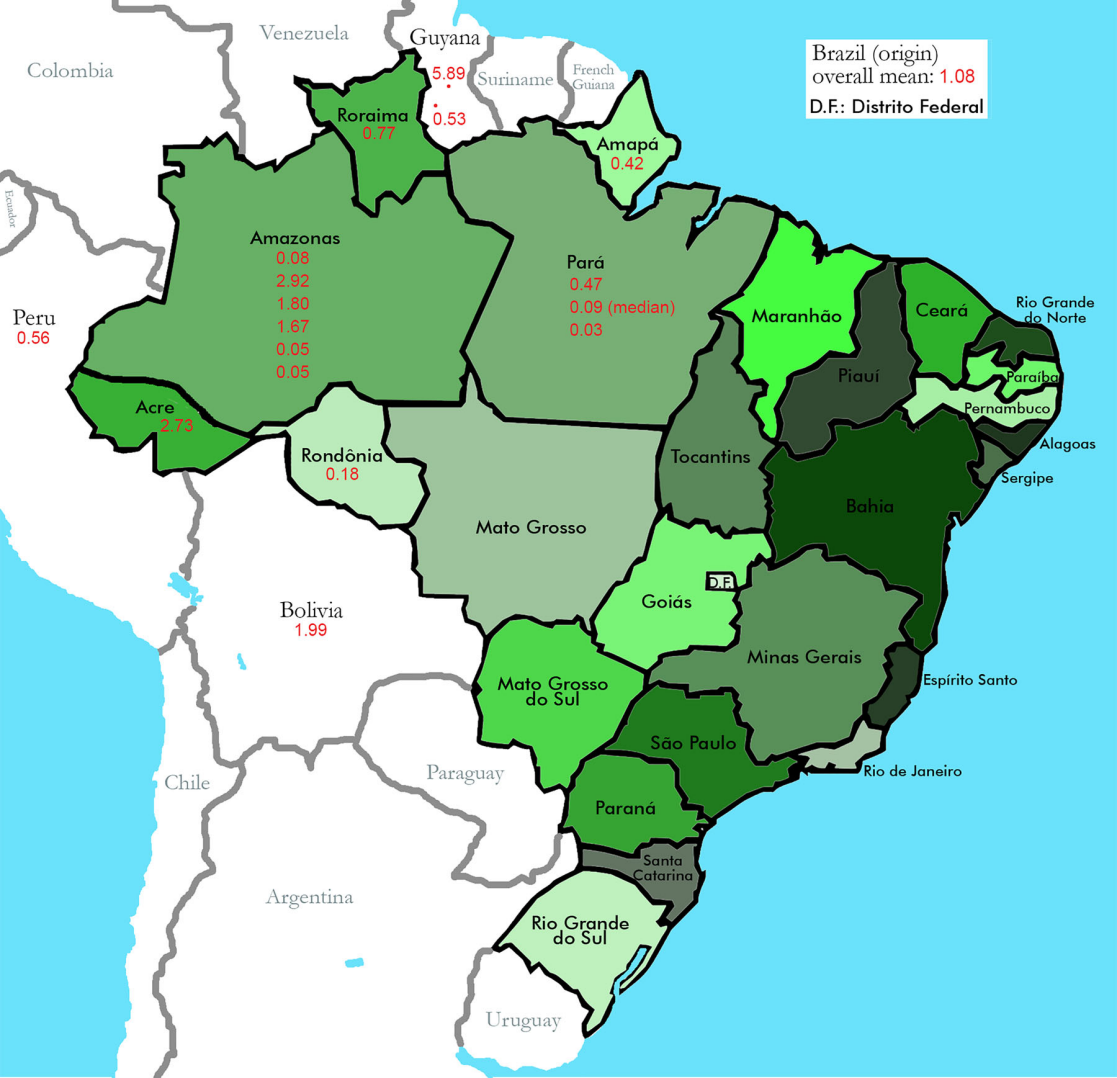
Barium levels in Brazil nuts from different regions of South America. Mean barium concentrations (mg/g) are presented.

The mean barium concentration based on data reported in the literature was 1.27 ± 0.19 mg/g (range: 0.03–5.89 mg/g; 24 publications; values: *n* = 43). Studies from Brazil showed a slight tendency toward lower mean barium concentrations in Brazil nuts (1.00 ± 0.18 mg/g; range: 0.03–2.73 mg/g: *n* = 22) compared to studies from the United States (US; 1.15 ± 0.28 mg/g; range: 0.10–1.99 mg/g; *n* = 7) and Europe (1.46 ± 0.36 mg/g; range: 0.07–2.92 mg/g; *n* = 9). A 1970s study reported mean barium levels of 3.21 ± 2.68 mg/g (*n* = 2) in Brazil nuts from Guyana and of 0.26 ± 0.07 mg/g (*n* = 2) in those from Singapore (Smith 1971). A 1980s study (Japan) found a barium level of 4.37 mg/g (*n* = 1). Apart from these two studies, all identified studies assessed barium levels in Brazil nuts obtained in Brazil, Europe, or the United States (Table 1). A sensitivity analysis using publication‐level means (instead of subgroup‐level means; Supporting Information 2) and a sensitivity analysis excluding the one study of defatted Brazil nuts (Supporting Information 3) largely confirmed these results. Figure 1 shows barium levels in Brazil nuts originating from different regions of South America.

## 6 Preliminary Upper Limits of Brazil Nut Intake Based on Barium Levels

Based on the mean barium concentration in Brazil nuts reported in the literature of 1.27 mg/g, consuming one Brazil nut (4–5 g) daily would result in a barium intake of about 5.1–6.4 mg/day (from Brazil nuts). For adults, this intake does not exceed the proposed tolerable daily intakes (TDIs) for long‐term barium exposure of 0.2 mg/kg body weight per day (mg/kg BW/day) (EC 2012; US EPA 2005; USDHH and ATSDR 2007) and 0.21 mg/kg BW/day (WHO 2016, 2022). However, one Brazil nut per day would exceed these TDIs for children weighing about 25–32 kg or less. For a 70‐kg adult, the TDI ranges from 14.0 mg/day (EC 2012) to 14.7 mg/day (WHO 2022). Consequently, consuming three Brazil nuts daily (12–15 g; ca. 15–19 mg barium) would exceed current TDIs. Recommendations for upper intake levels of Brazil nuts for adults, based on observed barium concentrations, are presented in Table 2.

**TABLE 2:** Suggested preliminary upper limits for Brazil nut intake based on barium content.

			Barium		
Region	*n*	Suggested max. intake	Approx. concentration (mg/g)	Approx. intake (mg/day)	TDI (mg/day)
**Brazil**	22	**3/day**	1.0	14	14–14.7
**Germany**	5	**1/day**	1.8	8	
**Guyana**	2	**6/week**	3.2	12	
**Japan**	1	**4/week**	4.4	11	
**Poland**	2	**2/day**	1.1	10	
**Singapore**	2	**11/day**	0.3	13	
**Spain**	1	**2/day**	1.4	12	
**Sweden**	1	**8/day**	0.4	14	
**USA**	7	**2/day**	1.2	10	
**Europe**	10		1.5	13	
**All studies**	44		1.3	12	

*Note*: Upper intake levels of Brazil nuts are based on not exceeding the barium TDI of 0.2–0.21 mg/kg body weight/day for a 70‐kg individual (Kowalczyk et al. 2022; WHO 2016). For individuals weighing < 70 kg, recommended amounts should be proportionally lower. A Brazil nut weight of 4.5 g was assumed. approx.: approximate; max. intake: maximum average intake of Brazil nuts per day or week; n: number of mean values; TDI: tolerable daily intake.

## 7 Previously Reported Barium Levels in Brazil Nuts

The present literature review found an overall mean barium level of 1.27 mg/g. In the 1960s, a barium level of no < 2–3 mg/g in Brazil nuts was reported (Mayneord 1960). Publications from the 21st century have reported individual barium levels as low as 0.002 mg/g (Cardoso et al. 2017) and maximum barium levels of 1.44 mg/g (Cardoso et al. 2017), 3 mg/g (Peana et al. 2021; US EPA 2005), and 4 mg/g (Lemire et al. 2010; Martinez‐Morata et al. 2023; Rayman 2008). These levels are similar to the maximum mean level found in the present review for Brazil nuts obtained in Brazil (2.73 mg/g; Table 1). A higher a mean barium level in Brazil nuts (5.89 mg/g) was reported for Brazil nuts from Rewa, Guyana (1970s; Table 1) (Smith 1971). The predominant reasons for the high barium levels in Brazil nuts appear to be soils rich in barium (hollandite), accumulation of barium by the Brazil nut tree (da Silva Junior et al. 2022; Penna‐Franca et al. 1968; USDHH and ATSDR 2007; WHO 1990), and efficient barium transport to the fruits (capsules) and the nuts (da Silva Junior et al. 2022). The tendency of the Brazil nut tree to accumulate barium may be influenced by tree genotype, and certain genotypes from the state of Pará (Brazil) appear to produce nuts with lower barium levels (da Silva Junior et al. 2022).

## 8 Barium and Health

Given the relatively high barium levels observed in this review, consuming two or three Brazil nuts per day may exceed the TDIs for barium (Table 2). However, the long‐term health effects of such intake levels remain unknown. Based on the literature data, the recommended intake levels (Table 2) would not result in a barium intake exceeding the TDIs. Notably, the TDIs for barium are based on studies of mice given barium chloride dihydrate dissolved in drinking water. From these studies, a benchmark dose 95% lower confidence limit (BMDL05) was derived, which was then divided by 300 (intra(Hiromoto et al. 1996)species variation: factor 10; interspecies variation: factor 10; database deficiencies: factor 3) (WHO 2016, 2022). Therefore, the applicability of these TDIs to barium intake from Brazil nuts in humans is highly uncertain.

In 2001, Dallas and Williams proposed an oral reference dose (a safe upper level) of 0.6 mg/kg BW/day as an alternative to current TDIs for barium (0.2–0.21 mg/kg BW/day) (Dallas and Williams 2001). However, this value was criticized and not accepted by the European Commission (EC 2012), while the current TDIs are widely supported (EC 2012; US EPA 2005; USDHH and ATSDR 2007; WHO 2022). Nevertheless, the current TDIs are generally considered conservative (EC 2012). The barium dose resulting in adverse effects in humans is unknown, and it remains highly uncertain whether exceeding the current TDIs through Brazil nut consumption poses a true health risk (Pearson and Ashmore 2020).

It has been suggested that barium ingestion from Brazil nuts may be harmless because the barium salts present are largely insoluble (Leonardos 1958; Silva Duarte et al. 2023) and barium absorption in the human intestine may be very low (Andrade et al. 1999; Gonçalves et al. 2009). The Agency for Toxic Substances and Disease Registry (ATSDR) has suggested gastrointestinal absorption of barium in humans to typically be in the range of <5%–30% of the administered dose (ATSDR 2007). However, multiple factors (e.g., calcium intake and food matrix) influence intestinal barium bioavailability (the percentage of barium absorbed) in humans (ATSDR 2007; Roselli et al. 2020), and not all barium salts exhibit the same bioavailability. For example, barium sulfate (barite, BaSO_4_) is commonly used in barium swallow studies to visualize the gastrointestinal tract (Khartade et al. 2020). In this context, intestinal absorption is typically minimal, and BaSO_4_ is generally considered safe for ingestion (Copeland et al. 2023; ICRP 1993). BaSO_4_ is also found in cosmetics and personal care products (Peana et al. 2021). Other barium salts such as barium chloride (BaCl_2_), barium carbonate (BaCO_3_), barium sulfide (BaS), barium oxide (BaO), barium acetate (Ba[C_2_H_3_O_2_]_2_), barium nitrate (Ba[NO_3_]_2_), and barium hydroxide (Ba[OH]_2_) have been described to exhibit considerably higher intestinal bioavailability, and cases of fatal barium intoxication have been reported (Ananda et al. 2013; Copeland et al. 2023; Schorn et al. 1991).

A substantial proportion of the barium in Brazil nuts may occur as BaSO_4_ (Gonçalves et al. 2009), particularly when barium levels are high, and possibly as barium selenate (BaSeO_4_), particularly when selenium levels are high (da Silva Junior et al. 2022). Similar to BaSO_4_ (Payen et al. 2011), bioavailability of BaSeO_4_ has been proposed to be very low (da Silva Junior et al. 2022). However, BaSO_4_ can potentially be toxic if intestinal barrier function is compromised, such as in individuals with colon cancer (Kravchenko et al. 2014; USDHH and ATSDR 2007). Moreover, it has been proposed that the low pH and high chloride concentration in the human stomach might increase the bioavailability of BaSO_4_ (ATSDR 2008; US EPA 2005). However, the International Commission on Radiological Protection (ICRP) does not classify BaSO_4_ among the acid‐soluble barium salts (which include barium acetate, carbonate, chloride, hydroxide, nitrate, and sulfide) that readily dissolve in gastric acid and are subsequently absorbed (ICRP 1993).

Data on the intestinal absorption of barium from Brazil nuts in humans appear to be limited to a recent randomized controlled trial (RCT) in Brazil (Silva Duarte et al. 2023) and a mass balance study from the 1980s in the United States, the latter cited by the World Health Organization (WHO) in this context (Lisk et al. 1988; WHO 2016).

The RCT from Brazil showed that consuming one Brazil nut (ca. 5 g) per day for 8 weeks did not affect plasma barium levels in women with obesity (*n* = 55) (Silva Duarte et al. 2023). However, the Brazil nuts in this study contained very low barium levels (ca. 0.05 mg/g), only about 4% of the average level found in the present review (1.27 [range: 0.03–5.89] mg/g). Therefore, the findings of this RCT may not be generalizable to Brazil nuts overall (or to Bolivian Brazil nuts, which may be higher in barium; Table 1).

In the mass balance study (the United States) (Lisk et al. 1988), a single participant (“man A”; age: 56 years; body weight: 68 kg) consumed a one‐time dose of about 92 g ground Brazil nuts, estimated to contain about 179.2 mg barium, that is, about 13 times the current TDI for a 68 kg person (ca. 14 mg Ba/day) (EC 2012; USDHH and ATSDR 2007; WHO 2016). The Brazil nuts purchased locally (presumably New York State), in‐shell, had a reported barium content of 1.95 mg/g. All feces and urine were collected for 6 days prior to and 15 days after ingestion. Each day, samples were weighed, mixed, and sub‐sampled. Urine samples were evaporated, feces samples freeze‐dried, and both were dry‐ashed and reignited prior to barium analysis. The limit of detection (LoD) for barium was reported to be quite low (0.01 µg/g (Lisk et al. 1988)) compared to the LoD of 0.1 µg/g provided to us by a laboratory in Germany (unpublished data). However, this discrepancy may be due to different formulas being used for calculating the LoD (Gegenschatz et al. 2022). Blood barium levels were not measured. During the 6 days following Brazil nut ingestion, it was observed that of the ingested barium (ca. 179.2 mg), about 9.09% (ca. 16 mg) and 0.81% (ca. 1 mg) were excreted in feces and urine, respectively, meaning 90.1% (ca. 161 mg) was not recovered in feces or urine (during the 6‐day assessment period). Based on this, the authors concluded that approximately 90% of the ingested barium was deposited in the participant's tissues (Lisk et al. 1988).

Notably, the article by Lisk et al. (1988) reporting this experiment describes two separate experiments, and the study design is not entirely clear. The first experiment (a selenium study) involved four participants, including “man A.” For this part, “man A” consumed 3 Brazil nuts/day (13.2 g/day) for 16 days (ca. 211 g total), followed by 6 Brazil nuts/day (26.4 g/day) for 27 days (ca. 713 g), totaling 924 g in 43 days.

In the second experiment (the barium study), “man A” consumed a single dose of 91.76 g Brazil nuts. Although this appears to be a separate experiment, the article does not explicitly state this nor specify the timing relative to the selenium study (e.g., whether there was a washout period). Hypothetically, errors in sub‐sampling feces and urine may have occurred, and the barium levels in the analyzed Brazil nuts may have differed substantially from those in the consumed nuts. Given these potential uncertainties, the results of this mass balance study (Lisk et al. 1988) should be interpreted with caution. Moreover, it has been suggested that absorption estimates based on balance studies are highly uncertain (ICRP 1993).

Furthermore, ICRP and the Environmental Protection Agency (EPA [USA]) cite an unpublished UK doctoral dissertation reporting a barium‐140 (BaCl_2_ in orange juice) absorption of 3%–16% in five female cancer patients with normal gastrointestinal function (ICRP 1993; US EPA 2005). Other human studies indicate barium absorption ranging from negligible to 60% (ICRP 1993). Thus, current knowledge of barium bioavailability and excretion from Brazil nuts in humans remains extremely limited. Well‐designed human studies employing modern analytical methods are needed to improve understanding.

It has been suggested that fecal and urinary barium levels reflect recent exposure (previous 3 days) for up to 2 weeks post‐ingestion, while whole blood barium may not be a reliable biomarker of exposure (Martinez‐Morata et al. 2023). Barium is thought to be cleared from circulation within 24 h, via excretion (typically >90% in feces; ca. 2%–5% in urine) or deposition in tissues, mainly bone (Lisk et al. 1988; WHO 2016).

The primary concern regarding excessive barium intake is thought to be kidney damage (which has been observed in mice in the laboratory given drinking water with high concentrations of barium chloride dihydrate) (WHO 2016). Evidence of a carcinogenic or genotoxic effect of barium is lacking (WHO 2022). Furthermore, none of the known symptoms of acute barium toxicity (e.g., vomiting, paresthesia, or convulsions [Kowalczyk et al. 2022]) have been described in association with Brazil nut consumption.

Excessive barium intake may increase cardiovascular disease (CVD) risk (Peana et al. 2021). However, several observational studies found an inverse relationship between barium intake and CVD risk (Duan et al. 2020; Elwood et al. 1974; Guo et al. 2022; Schroeder and Kraemer 1974; Zhu et al. 2020). Observational studies have shown inconsistent associations between barium intake and blood pressure (mild increases (Everson et al. 2021; T. Liu et al. 2021), decreases (Y. Liu et al. 2022; Zeng et al. 2022), or no association (Brenniman et al. 1981)). A 10‐week intervention study involving 11 men consuming high‐barium water (10 mg/L during the last 4 weeks) showed no effect on CVD risk markers (including blood pressure and cholesterol, apolipoprotein, and glucose blood levels) (WHO 2022; Wones et al. 1990). At present, any influence of moderately increased barium intake on CVD risk appears uncertain (Domingo‐Relloso et al. 2019; Duan et al. 2020; Guo et al. 2022; Kravchenko et al. 2014; Y. Liu et al. 2022; Navas‐Acien et al. 2005; Sowden and Stitch 1957; Swayze et al. 2021; Zhu et al. 2020).

While studies in the 1930s had suggested that human bone did not contain barium, this was disproven in the 1950s. The average adult human contains about 22 mg of barium (Lisk et al. 1988; Sowden and Stitch 1957), with 91–93% located in bone (Lisk et al. 1988; WHO 2016) and the remainder in soft tissues (Dallas and Williams 2001). A potential bone‐protective effect of barium has been proposed. A small study in the United States observed that lower barium levels in bone were associated with osteoporotic trabecular bone (Coyte et al. 2022). Additionally, a 10‐week intervention study with 11 men (United States) consuming high‐barium drinking water observed a trend toward increased serum calcium levels with higher barium exposure (Wones et al. 1990). Furthermore, a small post‐mortem study (United States) found that bones from patients who had received long‐term parenteral nutrition had significantly lower calcium but higher barium levels than control bones obtained during hip or knee replacement surgery (Galusha et al. 2021).

In addition, elevated barium/calcium ratios have been observed in the teeth of children exposed to high‐barium drinking water (ca. 10 mg/L) (WHO 2016). Barium might have a protective effect on teeth. A comparison of 21 villages in Papua New Guinea (1970s) found an inverse association between caries prevalence and barium levels in garden soils, vegetables, and drinking water (Barmes et al. 1970). Similarly, a cross‐sectional study in the United States (1980s) found that children in a town with high‐barium drinking water (ca. 8–10 mg/L) had less caries than those in a town with low barium water levels (<0.03 mg/L), with no other factor identified to explain this difference (Zdanowicz et al. 1987). Moreover, a recent study in the United States found lower barium levels in deciduous tooth dentine of children fed predominantly breast milk in the first year of life compared to those who predominantly received formula (Friedman et al. 2022).

A case‐control study from China reported an association between higher barium concentrations in placental tissues and an increased risk of orofacial cleft (Pi et al. 2019). However, higher barium exposure in this study may reflect greater exposure to air pollution sources (e.g., indoor coal or gasoline burning) (Nordberg et al. 2015; Pi et al. 2019). Beyond air pollution, other relevant sources of barium include certain work environments, living near a waste site or in areas with high barium water levels, children's toys, and food (ATSDR 2013; Kravchenko et al. 2014). Foods occasionally reported to contain high barium levels include dairy products, soy foods, cabbage, and nuts (Martinez‐Morata et al. 2023). However, in comparison to the barium levels in Brazil nuts (present review: 1.27 mg/g), the barium concentration in other foods is typically much lower: fish, milk, and yogurt (0.0001 mg/g); meat (0.0001–0.0004 mg/g); fruit (0.0003–0.0005 mg/g); vegetables (0.0003–0.0008 mg/g); eggs (0.0005 mg/g); cheese (0.0005–0.0009 mg/g); legumes (0.0005–0.0010 mg/g); cereal grains (0.0007–0.0016 mg/g); oil, butter, and margarine (0.0020–0.0030 mg/g); herbs and spices (0.0260 mg/g); breakfast cereals (0.0030 mg/g); and nuts (0.0040–0.1310 mg/g) (González‐Weller et al. 2013; Pearson and Ashmore 2020; Rose et al. 2010; WHO 2016). Typical dietary barium intake has been estimated to be in the range of about 0.30–3.15 mg/day (Choudhury and Cary 2001; González‐Weller et al. 2013; Kravchenko et al. 2014; Rose et al. 2010) or 0.004–0.045 mg/kg BW/day (Choudhury and Cary 2001; Rose et al. 2010). Therefore, Brazil nut consumption considerably increases barium intake.

The current WHO guideline value (upper limit) for barium in drinking water is 1.3 mg/L, which corresponds to 20% of the TDI (0.21 mg/kg BW) for a 60‐kg adult, assuming a drinking water intake of 2 L/day (WHO 2022). Earlier guideline values were lower (Brenniman et al. 1981), whereas the upper limit set by the EPA (USA) is 2 mg/L (Kravchenko et al. 2014). Typical barium levels in drinking water have been described to be <0.1 mg/L (WHO 2022). Table 3 summarizes aspects relating to barium intake and health.

**TABLE 3:** Relevant aspects related to Ba intake and health.

Ba occurrence and exposure			References
Typical Ba levels	Brazil nuts	5.1–6.4 mg per Brazil nut^a^	See Table 1
	Most foods	0.00 mg/g	González‐Weller et al. (2013); Pearson and Ashmore (2020); Rose et al. (2010); WHO (2016)
	Nuts in general	0.00–0.13 mg/g	
	Herbs/spices	0.03 mg/g	
	Drinking water	< 0.10 mg/L	WHO (2022)
Upper limits for Ba in drinking water		1.3–2.0 mg/L, 2 mg/L	Kravchenko et al. (2014), WHO (2022)
Typical Ba intakes		0.30–3.15 mg/day	Choudhury and Cary (2001); González‐Weller et al. (2013); Kravchenko et al. (2014); Rose et al. (2010)
Proposed bioavailability of different Ba salts in humans (uncertain)	Low	BaSO_4_, BaSeO_4_	Copeland et al. (2023); da Silva Junior et al. (2022); ICRP (1993)
	High	BaCl_2_, BaCO_3_, BaS, BaO, Ba(C_2_H_3_O_2_)_2_, Ba(NO_3_)_2_, Ba(OH)_2_	Ananda et al. (2013); Copeland et al. (2023); Schorn et al. (1991)
Main reasons for high Ba content in Brazil nuts		High barium soil levels, accumulation by the Brazil nut tree	da Silva Junior et al. (2022); Penna‐Franca et al. (1968); USDHH and ATSDR (2007); WHO (1990)
RCTs on Brazil nut intake and blood Ba levels		1 RCT (women in Brazil, *n* = 55)	Silva Duarte et al. (2023)
Potential effects of Ba on target tissues	Adverse	Kidneys, nervous system, cardiovascular system	Kowalczyk et al. (2022); Peana et al. (2021)
		Upper lip/palate (orofacial cleft)	Pi et al. (2019)
	Beneficial	Bones	Coyte et al. (2022); Galusha et al. (2021); Wones et al. (1990)
		Teeth	Barmes et al. (1970); Friedman et al. (2022); WHO (2016); Zdanowicz et al. (1987)

*Note*: The data in this table are generalizations and should be interpreted with caution.

^a^Assuming a weight of 4–5 g per Brazil nut; Ba: barium; RCT: randomized controlled trial.

## 9 Future Research

It would be useful for future studies to include a variety of producing countries (particularly Brazil, Bolivia, and Peru, to test the hypothesis that Brazil nuts from Bolivia are higher in barium) and to compare distinct forest zones and certified organic/conventional nuts. Future research may include data on soil type, local mineralogy, and the precise location of Brazil nut sampling, incorporating geospatial data (georeferencing). Geospatial risk maps may help guide the harvest and trade of Brazil nuts with lower barium levels. Future studies may also explore potential approaches to reducing barium levels in Brazil nuts (e.g., through increasing soil sulfur levels [de Souza Cardoso et al. 2023]) and assess their feasibility (Magalhães et al. 2012) in wild Brazil nut trees. Furthermore, human studies are needed to assess the bioavailability of barium from Brazil nuts. RCTs assessing the effects of regular Brazil nut consumption on markers of bone health (barium is a bone‐seeking element) as well as liver (Lisk et al. 1988) and kidney (the primary target of barium toxicity) function are warranted. Such studies could also clarify whether barium causally affects bone or dental health. Deciduous teeth can be collected non‐invasively and may be a useful medium for assessing barium exposure. A detailed discussion of barium in other foods or dietary patterns (Kaur et al. 2020; Shiraishi et al. 1994; USNRC 1999) and a discussion of other elements in Brazil nuts were beyond the scope of the present review. We recently published an overview of radium levels in Brazil nuts, which also suggests that consuming one Brazil nut daily is generally safe for adults (Koeder and Keller 2025).

## 10 Strengths and Limitations

The present review provides a global overview of published data on barium in Brazil nuts. However, some data may have been missed, particularly given the extensive gray literature on Brazil nuts in Portuguese (EMBRAPA et al. 1981). For one barium value, only the median (not the mean) was available (Lemire et al. 2010). Overall, the results of this review appear to be representative.

## 11 Conclusion

Brazil nuts are a unique food distinguished by their high barium content compared to other commonly available nuts. Although data are limited, a typical barium level in Brazil nuts of about 1.3 mg/g (95% confidence interval: 0.9–1.6 mg/g) can be assumed. This suggests that an average long‐term intake of one Brazil nut daily by adults generally does not exceed current TDIs and can therefore be considered safe. However, repeated assessments of locally purchased Brazil nuts are needed to develop more reliable and region‐specific intake recommendations. Current evidence remains insufficient to draw firm conclusions about clinically relevant health effects of barium exposure from Brazil nut consumption. When evaluating the safety of Brazil nuts, caution is warranted, and considerations related to planetary health should also be considered.

## Author Contributions

**Markus Keller**: conceptualization, writing – review and editing, project administration, supervision, resources. **Christian Koeder**: conceptualization, investigation, methodology, validation, formal analysis, project administration, data curation, writing – original draft, writing – review and editing.

## Conflicts of Interest

The authors declare no conflicts of interest.

## Supporting information




**Supplementary Materials**: jfds70643‐sup‐0001‐SuppMat.docx
