# Supplementary material for: Barium Levels in Brazil Nuts: A Global Review of the Literature
Source: J Food Sci. 2025 Oct 31;90(11):e70643. doi: 10.1111/1750-3841.70643 (PMC12576310; doi:10.1111/1750-3841.70643)
Supplement: Supplementary file 1 — Supplementary Materials: jfds70643‐sup‐0001‐SuppMat.docx [file JFDS-90-0-s001.docx]

*Supplementary material*

Christian Koeder, Markus Keller: **Barium levels in Brazil nuts: a global review of the literature**

# Supplementary information 1

**Comments on Table 1: publications included and excluded**

## Included in the present literature review:

-- da Silva Junior et al. 2022 (DOI: 10.1016/j.jfca.2021.104329): the mean barium values (with standard deviations) were obtained from the first author.

-- Moraes de Brito et al. 2019 (DOI: 10.1016/j.lwt.2019.108383): the barium values in Table 1 of the present literature review (0.31 mg/g) are the mean of values 2, 3, and 4 in their Table 4.

-- Welna et al. 2014 (DOI: 10.1080/19440049.2014.880134) reported four barium values for different sample preparation procedures (in their Table 1). In the present literature review, we report the first of this value (P1; based on microwave-assisted digestion in HNO3 with H2O2) as this appears to be the most reliable value (Welna et al. 2014).

-- Lemire et al. 2010 (DOI: 10.1016/j.scitotenv.2010.05.022): the barium value reported by Lemire et al. 2010 (0.09 mg/g) is the median, not the mean (the mean was not documented and not available anymore; the first author does not have access to the database anymore).

-- Gonçalves et al. 2009 (DOI: 10.1590/S0103-50532009000400020) reported ten barium values (in their Table 4: Maranhão: 1.77 and 1.87; São Paulo: 2.08 and 2.07; Pará [unshelled)]: 0.89 and 0.91; Pará [unshelled in capsule]: 0.86 and 0.89; Pará [shelled]: 1.91 and 1.95 [mg/g]). The barium values in Table 1 of the present literature review are the five means of each of these five value pairs.

-- Parekh et al. 2008 (DOI: 10.1016/j.jfca.2007.12.001) reported barium values for four locations: Brazil, Bolivia, Peru as well as an unknown location. This fourth sample of Brazil nuts (unknown location) was labeled ‘‘Product of the USA’’ but as there is no commercial production of Brazil nuts in the United States, Parekh et al. (reasonably) assumed that this must have been a labeling error. In their article, they referred to the origin of this fourth sample as northern South America (NSA). It can be reasonably assumed that practically all commercially available Brazil nuts originate from the Amazon rainforest, which is located in the northern (i.e., the “northern half” of) South America. Several authors citing Parekh et al. have misinterpreted ‘‘northern South America’’ to mean probably Colombia or Venezuela (i.e., the very north of South America).

-- Furr et al. 1979 (DOI: 10.1007/BF01685442): the barium value shown in Table 1 of the present literature review (1.71 mg/g) was estimated based on a concentration of 1.764 mg/g dry weight reported by Furr et al. and an assumption of 3.4% water content in Brazil nuts.

-- Penna França et al. 1968 reported 15 barium levels in Brazil nut ash (the mean of the 15 barium values in their Table 1: 52.35 mg/g ash). Based on an estimate of 3.37% ash and on the 15 ash values for Brazil nuts in their Table 2, we estimated the mean barium level to be 1.76 mg/g wet weight.

-- Seaber 1933 (DOI: 10.1039/an9335800575): the barium value in Table 1 of the present literature review (1.74 mg/g) is the mean of the 17 values reported by Seaber. The Brazil nuts in the study by Seaber do not appear to have been purchased at a retail store (Seaber [1933]: “Brazil nuts from different supplies”). Some of the Brazil nuts were reported to originat from Pará and Amazonas (“Manaos”, i.e., Manaus), while others were from an unknown location. The author (Seaber) appears to be from the United Kingdom.

## Excluded from the present literature review:

-- Benzing et al. 2022 (DOI: 10.1016/j.jfca.2022.104533) did not report any original data regarding barium in Brazil nuts.

-- Kluczcovski et al. 2020 (DOI: 10.5296/jas.v8i2.17185) stated that barium was also analyzed but no results for barium are reported in their article.

-- Macan et al. 2020 (DOI: 10.1080/01480545.2020.1808667) did not report any original barium data.

-- Armelin et al. 2017 (https://inis.iaea.org/collection/NCLCollectionStore/_Public/49/009/49009613.pdf; last accessed 17 July 2025) reported the same barium value reported in Armelin et al. 2019.

-- Poddalgoda et al. 2017 (DOI: 10.1016/j.yrtph.2017.03.022) did not report any original data regarding barium levels in Brazil nuts.

-- Kelmer et al. 2015 (DOI: 10.5935/0103-5053.20150177) did not report any barium levels in Brazil nuts.

-- Yin et al. 2015 (DOI: [10.3967/bes2015.063](https://doi.org/10.3967/bes2015.063)) did not report any barium levels in Brazil nuts.

-- da Silva 2014 (master thesis, Brazil, DOI: 10.11606/D.11.2014.tde-05012015-113355) did not report any barium levels in Brazil nuts.

-- Nordberg et al. 2014 (Handbook on the toxicology of metals. Cambridge: Academic Press. 2014) reported a barium range in Brazil nuts of 1500–3000 mg/kg, citing Moffett et al. 2017.

-- Moffett et al. 2007 (Toxicological profile for barium and barium compounds. Report. Agency for Toxic Substances and Disease Registry; 2007, <https://www.atsdr.cdc.gov/toxprofiles/tp24.pdf>; last accessed 17 July 2025) reported the same range for barium in Brazil nuts as Underwood 1977 (3000–4000 ppm), citing Beliles 1979 (Beliles RP. 1979. The lesser metals. In: Oehme FW, ed. Hazardous and toxic substances. Vol. 2. Toxicity of heavy metals in the environment. Parts 1 and 2. New York, NY: Marcel Dekker, Inc., 547­ 615.), which we had no access to.

-- Marx et al. 2000 (DOI: [10.17660/ActaHortic.2000.531.39](https://doi.org/10.17660/ActaHortic.2000.531.39)) reported a barium level of 1.5 mg/g in Brazil nuts. It is not explicitly stated whether this is an original value or a literature value, but it appears to be the value reported by Andrade et al. 1999 (whom Marx et al. 2000 cite).

-- Tinggi and Reilly 2000 (conference paper): Concentrations of selenium and other major elements (barium, calcium, magnesium, potassium and phosphorus) in Brazil nuts in Brisbane, Queensland. Selenium 2000, Venezia, October 1–5, which was cited by Welna et al. 2008. We were unable to obtain this paper from any of the authors.

-- Reilly 1999 (DOI: 10.1111/j.1467-3010.1999.tb00905.x) reported no original barium levels in Brazil nuts but cited Lisk et al. 1988.

-- Bois-Grossiant & Tan 1995 (DOI: 10.1016/0955-7997(95)00063-1) did not report any barium levels in Brazil nuts.

-- Mertz 1986 (Merz W. Trace elements in human and animal nutrition. Fifth edition. Volume 2. Academic Press. 1986, page 419) cites Robinson and Edgington 1945 (W. O. Robinson and G. Edgington, Soil Sci. 60, 15 (1945); https://journals.lww.com/soilsci/Citation/1945/07000/MINOR_ELEMENTS_IN_PLANTS,_AND_SOME_ACCUMULATOR1.3.aspx; last accessed 17 July 2025), which we had no access to (“Robinson and co-workers (213,214) […] found an unusually high level of barium in Brazil nuts (range 700–3200 μg/g) […]”), and Robinson et al. 1950 (Robinson, W. O., Whetstone, R. R., and Edgington, G. (1950) U.S., Dep. Agric, Tech. Bull. 1013, 1–36) who only cited Wagner 1936 and Seaber 1933.

-- Calabrese et al. 1985 (DOI: 10.1146/annurev.pu.06.050185.001023) reported the same range for barium in Brazil nuts as Underwood 1977 (3000–4000 ppm).

-- Lott & Buttrose 1977 (DOI: [10.1139/B78-245](https://doi.org/10.1139/B78-245)) assessed but did not report barium levels in Brazil nuts.

-- Underwood 1977 (Underwood E. Trace elements in human and animal nutrition. Fourth edition. Academic Press. 1977, page 435): they reported ranges of barium in Brazil nuts (3000–4000 ppm), citing Robinson and Edgington 1945 (W. O. Robinson and G. Edgington, Soil Sci. 60, 15 (1945); https://journals.lww.com/soilsci/Citation/1945/07000/MINOR_ELEMENTS_IN_PLANTS,_AND_SOME_ACCUMULATOR1.3.aspx; last accessed 17 July 2025), which we had no access to, and Seaber 1930 (reference 70: W. Seaber, Analyst 58, 575 (1930 [the year should be 1933])) which is included in the present literature review (Seaber 1933, DOI: [10.1039/AN9335800575](https://doi.org/10.1039/AN9335800575)).

-- Schroeder et al. 1972 (DOI: 10.1016/0021-9681(72)90150-6) reported the same range for barium in Brazil nuts as Underwood 1977 (3000–4000 ppm).

-- Bowen 1966 (Bowen HJM. Trace elements in biochemistry. New York: Academic Press. 1966, page 176) reported no original data regarding barium in Brazil nuts: They stated that barium levels in Brazil nuts can be “up to 4000 ppm”, citing Seaber 1933 and Wagner 1936.

-- Leonardos 1958 (Leonardos, Othon: Sobre a radioatividade das castanhas do Para, Annaes da Academia Brasileira de Ciencia, Volume 30(4), page LI-LII. 1958; http://memoria.bn.br/DocReader/DocReader.aspx?bib=158119&Pesq=leonardos&pagfis=11661; last accessed 17 July 2025) reported no original barium levels in Brazil nuts but cited Seaber 1933.

-- Robinson et al. 1950 (https://babel.hathitrust.org/cgi/pt?id=uiug.30112019331559&view=1up&seq=3; last accessed 17 July 2025) reported ranges for barium oxide citing Wagner 1936 and Seaber 1933.

# Supplementary information 2

**Sensitivity analyses for barium using publication-level means (instead of subgroup-level means)**

Sensitivity analyses using publication-level means (instead of subgroup-level means) largely confirmed the results: the mean barium levels changed from 1.27 ± 0.19 mg/g (subgroup-level means: n = 43) to 1.32 ± 0.21 mg/g (publication-level means: n = 25); from 1.00 ± 0.18 mg/g (subgroup-level means, Brazil: n = 22) to 0.96 ± 0.19 mg/g (publication-level means, Brazil, n = 12); from 1.15 ± 0.28 mg/g (subgroup-level means, USA: n = 7) to 1.38 ± 0.27 mg/g (publication-level means, USA, n = 4); and from 1.46 ± 0.36 mg/g (subgroup-level means, Europe: n = 9) to 1.38 ± 0.41 mg/g (publication-level means, Europe, n = 6).

# Supplementary information 3

**Sensitivity analyses excluding studies that analyzed barium levels in defatted or partially defatted Brazil nuts**

Sensitivity analyses excluding the one study that analyzed barium levels in defatted Brazil nuts (Poland: Welna and Szymczycha-Madeja 2014) largely confirmed the results: mean barium levels (subgroup-level means) changed from 1.27 ± 0.19 mg/g (n = 43) to 1.25 ± 0.19 mg/g (n = 42); (publication-level means) from 1.32 ± 0.21 mg/g (n = 25) to 1.29 ± 0.21 mg/g (n = 24); (subgroup-level means, Europe) from 1.46 ± 0.36 mg/g (n = 9) to 1.36 ± 0.39 mg/g (n = 8); and (publication-level means, Europe) from and from 1.38 ± 0.41 mg/g (n = 6) to 1.21 ± 0.45 mg/g (n = 5).
